# Dysmenorrhea and its associated symptoms among girls with special needs in Upper Egypt: an exploratory study

**DOI:** 10.1186/s42506-025-00198-8

**Published:** 2025-10-02

**Authors:** Doaa M. Osman, Shimaa A. Khalaf, Heba M. Mohamed, Reda R. Ali, Fatma R. Khalaf

**Affiliations:** 1 Public Health and Community Medicine Department, Faculty of Medicine, Assiut University, Asyut, Egypt; 2 Family and Community Health Nursing Department, Faculty of Nursing, Assiut University, Asyut, Egypt; 3 Maternal and Newborn Health Nursing Department, Faculty of Nursing, Asyut University, Asyut, Egypt

**Keywords:** Adolescents, Disability, Dysmenorrhea, Egypt

## Abstract

**Background:**

Dysmenorrhea is a highly prevalent gynecological problem that may compromise the girls’ quality of life. Disabled girls might experience menstruation both differently and more negatively compared to non-disabled girls.

The current study aimed to investigate the prevalence of dysmenorrhea and its associated symptoms amongst disabled girls.

**Methods:**

A cross-sectional study was conducted among 93 adolescent girls with disabilities. An interviewer questionnaire was used to inquire about personal characteristics, menstrual history, knowledge and attitudes of dysmenorrhea, symptoms that coincide with menstruation, and methods used by girls to manage their menstrual pain. WaLIDD scale was used to assess pain intensity during menstruation.

**Results:**

About 97% of the studied disabled girls suffered from moderate or severe degrees of menstrual pain. Using methods to mitigate the pain was reported by 72%. Herbal drinks and analgesics were the most frequently used methods. School absenteeism because of dysmenorrhea was reported by 36%. Most of the girls (87.1%) had a poor knowledge level. The most frequent disabling/severe symptoms that coincide with dysmenorrhea were general aches, dizziness, and fatigue. Increased perception of symptoms coinciding with menstruation was a significant predictor for severe dysmenorrhea (AOR = 3.279, 95%CI = 1.028:1.088).

**Conclusion and recommendations:**

Most disabled girls suffer from moderate to severe dysmenorrhea. Increased symptoms associated with menstruation positively affect severe dysmenorrhea perception. Girls with disabilities need better access to menstrual health education. Tailored reproductive health programs should be provided for blind and deaf girls for assurance and proper practice to manage symptoms associated with menstruation, especially pain, to mitigate their disabling impact.

## Introduction

Globally, there are an estimated 1.3 billion significantly disabled people, forming 16% of the world’s population [1]. Hearing loss and blindness are major contributors to global disability [2, 3]. More than 5% of the world’s population requires rehabilitation for hearing loss [4]. The global estimate of blind people is 43.3 million, and 295 million are estimated to have moderate to severe vision impairment [5].

The greatest burdens of hearing loss and blindness are concentrated in economically disadvantaged countries [3, 5]. In Egypt, a national household survey estimated the prevalence of hearing loss at 16%, which is higher than in many other countries [6], and approximately 1 million people are blind, while 3 million are visually impaired [7].

Disabled people often experience stigmatization, disregard, and violations of their rights, dignity, and autonomy. Their health and reproductive rights are significantly neglected. Disabled girls and women face the double burden of having an impairment and being female [8]. The physical pubertal transition is a complicated time for most adolescents and their families, and it is even more challenging for teenagers with disabilities [9].

Menstrual health is a fundamental component of sexual and reproductive health [10]. Menstruation is often more challenging for girls and women with disabilities. Disabled girls experience menarche and menstruation both differently and more negatively compared to non-disabled girls. In addition, the menstrual health needs of disabled girls are poorly understood and addressed, exposing them to negative consequences for their health and quality of life [8].

Dysmenorrhea is the most common gynecologic problem. It is defined as pain during the menstrual cycle, often located in the lower abdomen and radiating to the back and inner thighs [11]. Dysmenorrhea has adverse impacts on the lives of female adolescents. It is the main cause of recurrent short-term absenteeism from school or work [12].

Arabic adolescent girls often have limited knowledge regarding self-care during menstruation and dysmenorrhea. Moreover, feelings of shyness and cultural taboos may prevent them from reporting their symptoms or seeking medical advice [13]. The negative experience of dysmenorrhea might be more pronounced among disabled adolescents [14].

Several factors have been reported to be associated with a higher risk of primary dysmenorrhea, including early menarche, irregular menstrual cycles, heavy blood flow, a positive family history, smoking, regular consumption of caffeinated beverages, nulliparity, emotional problems, and low levels of physical activity [15–17].

This study aimed to investigate the prevalence of dysmenorrhea and its associates among blind and deaf girls in Upper Egypt.

## Methods

### Study setting and design

A cross-sectional study design was applied. The study was conducted in El Noor and El Amal schools in Asyut Governorate. Asyut is one of the largest governorates in Upper Egypt. It Lies on the banks of the Nile and is located approximately 320 km south of Cairo Governorate with an estimated total population of 5,086,614 [18].

El Noor and El Amal schools are the only available schools for the blind and deaf in Asyut Governorate. They are governmental schools supervised by the Egyptian Ministry of Education. Data were collected during the academic year 2021/2022.

### Study population

The total number of students in Al Amal and El Noor schools was 126 and 250 female students, respectively.

**Inclusion and exclusion criteria**

All girls from both schools who had undergone puberty and agreed to participate in the study were recruited. The study included only girls who had menstrual cycle and had their menarche six months or more before the data collection process. None of the studied disabled girls had a cognitive impairment, as El Noor and El Amal schools assess the intelligence quotient (IQ) for all students before enrollment. They do not accept any girl with an IQ score of less than 75. The studied population consisted of 93 deaf and blind girl.

### Data collection procedure and the study tool

Data were collected using an anonymous, interviewer-administered questionnaire. The questionnaire was designed in English, based on the original language of the referenced scales.

Forward and backward translations into Arabic were performed by independent researchers. All translations were reviewed by a linguistic specialist and a gynecologist. The Arabic version of the questionnaire was designed in the Egyptian Arabic dialect, using simple language appropriate for adolescents.

To ensure clarity, the questionnaire was pilot tested on 10 non-disabled girls. No modifications were necessary following the test.

For deaf girls, data were collected in Egyptian Sign Language by the social specialist et al. Amal school, after being trained in data collection procedures. For blind girls, the researchers themselves conducted the interviews. They asked each question orally and recorded the responses.

The questionnaire included the following sections:

1. Sociodemographic characteristics: age, residence, parents’ education, and occupation.
2. Menstrual characteristics: age at menarche, cycle length and regularity, duration of menses, number of pads used during the first three days, perceived bleeding amount (deaf girls only), family history of dysmenorrhea [16, 19], and whether pain severity varies by season [20].
3. Pain management methods: bed rest, heating pad, exercise, massage, herbal drinks, analgesics, or no method [20].
4. Negative impacts of dysmenorrhea: poor concentration, impaired personal relationships, limited exercise, missing school or exams, clinic visits or hospitalization due to menstrual pain [20, 21].
5. Dysmenorrhea assessment:
  The WaLIDD instrument, developed by Aníbal A. Teherán and colleagues in 2018, permits the identification of women with dysmenorrhea and predicts the need for medical leave. It was validated among university students and showed good internal consistency and correlation with common pain rating scales.
  The scale assesses four aspects: number and location of pain, number of days in pain, disability level, and pain intensity (via a modified Wong–Baker scale). Each item is scored from 0 to 3, with a total possible score of 0–12. Dysmenorrhea severity was categorized as follows: 0 = none, 1–4 = mild, 5–7 = moderate, and 8–12 = severe [22]. The Cronbach's alpha in this study was 0.64.
6. Associated symptoms during menstrual flow: A symptom checklist was constructed based on prior literature [11, 23, 24]. Girls rated 19 symptoms (e.g., general ache, backache, headache, nausea, breast tenderness, anxiety, mood swings) on a 3-point scale: 0 = no, 1 = mild/moderate, 2 = severe/disabling. The total symptom score was calculated per participant. Internal consistency was high (Cronbach's alpha = 0.818).
7. Attitude toward menstruation:
  Measured using the Menstrual Attitude Questionnaire (MAQ), developed by Jeanne Brooks-Gunn and Diane N. Ruble [25]. This multidimensional tool captures personal beliefs and reactions to menstruation, using five domains: menstruation as debilitating, bothersome, natural, predictable, and having no effect. Items are rated on a 7-point Likert scale from 1 (strongly disagree) to 7 (strongly agree). Negative items were reverse-scored as guided. Scores for each domain were calculated as the average of item scores. Internal consistency was good (Cronbach's alpha = 0.742).
8. Knowledge of dysmenorrhea:
  Measured using a 17-item scale developed by the authors (based on 12, 26). Items assessed knowledge of dysmenorrhea definition, types, its relationship with a higher possibility of ovulation and fertility, risk factors, and pain management. Responses were scored as: correct = 1, incorrect or “don’t know” = 0.
  Knowledge level was categorized: Poor: <50%, Fair: 50–75%, and Good: ≥75%
  Cronbach's alpha = 0.58. Girls were also asked about sources of menstrual information (mother, sister, friends, teachers).

### Statistical analysis

Data were analyzed using IBM SPSS version 26. Categorical variables are presented as frequencies and percentages; numerical variables are shown as means and standard deviations or standard errors.

Internal consistency for all scales was evaluated and found to be satisfactory [26].

To identify factors associated with severe dysmenorrhea, bivariate analysis was performed using the Chi-square test and either the Student’s t-test or Mann–Whitney test.

A binary logistic regression model was constructed, with the dependent variable coded as:

- 1 = severe dysmenorrhea
- 0 = mild/moderate dysmenorrhea

Predictors included significant variables from the bivariate analysis, along with age. Continuous predictors were: age, associated symptoms score, and the disabling domain score from the MAQ. Family history was treated as a categorical variable (reference = no history).

A *p*-value of < 0.05 was considered statistically significant. Odds ratios and 95% confidence intervals were reported. Visualizations were prepared using Microsoft Excel.

## Results

Table 1 shows the socio-demographic data of disabled girls in Asyut City. The participants’ ages ranged from 13 to 22 years, with a mean of 18.03 ± 2.257 years. More than half (51.6%) of the sample were aged 15–18 years, and 43% were aged 19 or older. Deaf girls accounted for approximately 80% of the sample. Most students (92.5%) were urban residents. Regarding parents’ education, only 18.3% of fathers had university or postgraduate education, while 53.8% of mothers were either illiterate or could only read and write. More than half (51.6%) of fathers were unemployed or worked in unskilled jobs, and the majority of mothers (94.6%) were housewives.

**Table 1:** Socio-demographic data of studied disabled girls in Asyut City, Upper Egypt (Academic year 2021/2022)

Socio-demographic variables	No. (93)	%
**Age: (years)**		
< 15	5	5.4
15- 18	48	51.6
≥ 19	40	43.0
Mean ± SD (Range)	18.03 ± 2.257 (13:22)	
**Type of disability**		
Blindness	19	20.4
Deafness	74	79.6
**Family residence:**		
Rural	7	7.5
Urban	86	92.5
**Father education:**		
Illiterate/ read and write	27	29.1
Basic education	19	20.5
Secondary	30	32.3
University / Post university	17	18.3
**Mother education:**		
Illiterate/ read and write	50	53.8
Basic education	15	16.2
Secondary	20	21.5
University / Post university	8	8.7
**Father occupation:**		
Unemployed	7	7.5
Unskilled worker	41	44.1
Skilled worker / Farmer	21	22.6
Employee	16	17.2
Professional	8	8.6
**Mother occupation:**		
Housewife	88	94.6
Skilled worker	2	2.2
Employee	3	3.2
**Weekly drinking of coffee**		
Yes	66	71
No	27	29
**Weekly drinking of coffee (*n* = 66)**		
1–3	52	78.8
4–7	11	16.6
≥ 8	3	4.5

Table 2 describes menstrual characteristics among the studied girls. The mean age at menarche was 12.70 ± 1.247 years, and approximately 61% of students reached puberty at age 13 or older. The majority of girls were prepared for menarche. More than one-third reported having irregular menstrual cycles. An increase in menstrual pain severity during cold weather was reported by 14%. A positive family history of dysmenorrhea was reported by 72% of the students.

**Table 2:** Menstrual characteristics of the studied disabled girls in Asyut City, Upper Egypt (Academic year 2021/2022)

Menstrual variables	No. (93)	%
**Age at menarche in years**		
9–12	36	38.7
≥ 13	57	61.3
Mean ± SD (Range)	12.70 ± 1.247 (9:16)	
**Being informed on menstruation for girls menarche:**		
Yes	79	84.9
No	14	15.1
**Cycle regularity**		
Regular	60	64.5
Irregular	33	35.5
**Bleeding amount (*n* = 74):**		
Drops /mild/	2	2.7
Average	42	56.8
Heavy	28	37.8
Heavy with clots	2	2.7
**Cycle length:**		
21 days or less	8	8.6
21 to 28 days	45	48.4
29—35 days	22	23.7
˃ 35	18	19.4
**No. of pads used daily in the first three days:**		
≤ 2	63	67.7
3–4	29	31.2
˃ 4	1	1.1
Mean ± SD (Range)	2.23 ± 0.74 (1:5)	
**Pain severity change depending on the weather:**		
Yes, in the cold season	13	14.0
No	80	86.0
**Family history**		
Yes	67	72.0
No	26	28.0

Table 3 presents the intensity of menstrual pain using the WaLIDD scale, management methods, and impacts on daily activities. More than half of the participants (54.8%) reported severe menstrual pain, while nearly 42% experienced moderate pain. None of the girls reported an absence of dysmenorrhea. The mean WaLIDD score was 7.79 ± 1.75.

**Table 3:** Dysmenorrhea severity based on WaLIDD scale, the disabled girls’ management of menstrual pain and its impact on their activities in Assuit City, Upper Egypt (Academic year 2021/2022)

Variables	No. (93)	%
**Dysmenorrhea level based on the WaLIDD scale**		
Mean ± SD (range)	7.79 ± 1.75(3–11)	
**Grades of dysmenorrhea based on the WaLIDD scale**		
No dysmenorrhea	0	0.0
Mild	3	3.2
Moderate	39	41.9
Severe	51	54.8
**Management of dysmenorrhea**		
Yes	67	72.0
No	26	28.0
**Types of management usage **^**a**^**(*n* = 67)**		
Herbal drinks	66	98.5
Analgesic	13	19.4
Exercise	3	4.5
Massage	1	1.5
Warm pad	1	1.5
**Visited a public/private clinic for this pain**		
Yes	11	11.8
No	82	88.2
**Previously hospitalized because of menstrual pain**		
Yes	4	4.3
No	89	95.7
**Missed a school day because of your menstrual pain**		
Yes	34	36.6
No	59	63.4
**Missed an exam because of the menstrual pain**		
Yes	1	1.1
No	92	98.9
**Effect on personal relationships**		
Yes	8	8.6
No	85	91.4
**Effect on exercise**		
Yes	64	68.8
No	29	31.2

^a^More than one answer had been reported

Seventy-two percent of girls used multiple methods to relieve menstrual pain. The most commonly used method was herbal drinks (98.5%), followed by analgesics (19.4%). Clinic visits for pain relief were reported by 11.6%, and 4.3% of girls had been hospitalized due to menstrual pain.

Over one-third (36.6%) of girls missed school days due to menstrual pain. Dysmenorrhea negatively affected physical activity in 68.8% of cases and personal relationships in 8.6%.

Table 4 shows knowledge, attitudes toward menstruation, and severity of associated symptoms. Most girls (87.1%) had a poor level of knowledge, with a mean score of 6.76 ± 0.159. The most commonly reported sources of knowledge were mothers (88.2%), friends (83.9%), and teachers (64.5%).

**Table 4:** Knowledge scale, menstrual attitude scale, and associated symptoms score among studied Egyptian disabled girls in Asyut City, Upper Egypt (Academic year 2021/2022)

Variables	No.(93)	%
**Knowledge of dysmenorrhea**		
Poor	81	87.1
Fair	12	12.9
Good	0	0
Mean ± SD (Range)	6.76 ± 0.159 (3–10)	
**Source of knowledge on dysmenorrhea**		
Mother	82	88.2
Sister	30	32.3
Friends	78	83.9
Teachers	60	64.5
Internet	3	3.2
**Menstrual Attitude Scale (Mean** ± **SD)**		
Menstruation as a disabling score	5.227 ± 0.861	
Menstruation as a bothersome score	4.382 ± 0.730	
Menstruation as a natural event score	5.002 ± 0.578	
Anticipation of the onset of menstruation score	4.870 ± 0.773	
Denial of any effect of menstruation score	2.403 ± 0.816	
**Associated symptoms score: Mean** ± **SD (range)**	21.58 ± 6.19 (6–32)	

The mean score for severe symptoms associated with menstruation was 21.58 ± 6.19. The most frequently reported severe symptoms were general aches, dizziness, fatigue, suffocation, backache, and headache. The least frequently reported symptoms included weight gain, irritability, mood swings, and skin disorders.

Table 5 presents correlates of severe dysmenorrhea. A significant association was found with family history: 62.7% of those with severe dysmenorrhea had a positive family history, compared to 37.3% in the mild/moderate group (*P* = 0.015).

**Table 5:** Correlates of severe dysmenorrhea among the studied Egyptian disabled girls in Asyut City, Upper Egypt (Academic year 2021/2022)

Variables	Mild/ moderate dysmenorrhea	Severe dysmenorrhea	*P* value
**Age (Mean ± SD)**	17.64 ± 2.20	18.35 ± 2.28	0.149
**Disability type**			
Blindness	10(52.6%)	9(47.4%)	0.463
Deafness	32(43.2%)	42(56.8%)	
**Father education**			
Illiterate/ Read and write	15(55.6%)	12(44.4%)	0.138
Basic education	5(26.3%)	14(73.7%)	
Secondary / University or more	22(46.8%)	25(53.2%)	
**Mother education**			
Illiterate/ Read and write	23(46.0%)	27(54.0%)	0.236
Basic education	4(26.7%)	11(73.3%)	
Secondary / University or more	15(53.6%)	13(46.4%)	
**Father occupation**			
Unemployed/ skilled-unskilled worker	30(43.5%)	39(56.5%)	0.580
Employee/ professional	12(50.0%)	12(50.0%)	
**Mother occupation**			
Work	3(60.0%)	2(40.0%)	0.655
Housewife	39(44.3%)	49(55.7%)	
**Age at menarche (Mean ± SD)**	12.64 ± 1.46	12.76 ± 1.05	0.532
**Preparation for menarche**			
Yes	34(43.0%)	45(57.0%)	0.238
No	8(57.1%)	6(42.9%)	
**Cycle regularity**			
Regular	27(45.0%)	33(55.0%)	0.966
Irregular	15(45.5%)	18(54.5%)	
**No. of pads (Mean ± SD)**	2.12 ± 0.80	2.33 ± 0.68	0.112
**Family history**			
Yes	25(37.3%)	42(62.7%)	0.015
No	17(65.4%)	9 (34.6%)	
**Associated symptoms score (Mean ± SD)**	18.79 ± 5.66	23.88 ± 5.69	*P* < 0.001*
**Knowledge score (Mean ± SD)**	6.76 ± 1.61	6.77 ± 1.49	0.850
**Menstrual Attitude Scale (Mean ± SD)**			
Disabling score	5.02 ± 0.92	5.40 ± 0.78	0.041*
Bother score	4.30 ± 0.80	4.46 ± 0.67	0.426
Natural event score	5.09 ± 0.69	4.93 ± 0.46	0.414
Anticipation score	4.81 ± 0.78	4.92 ± 0.77	0.441
Denial score	2.55 ± 0.81	2.28 ± 0.81	0.107

*statistically significant

The mean scores for associated symptoms and the disabling domain of the Menstrual Attitude Scale (MAS) were significantly higher in the severe dysmenorrhea group (23.88 ± 5.69 and 5.40 ± 0.78, respectively) than in the mild/moderate group (18.79 ± 5.66 and 5.02 ± 0.92, respectively) (*P* < 0.001).

Severe dysmenorrhea was not significantly associated with any other personal or menstrual characteristics, knowledge scores, or MAS subdomains other than the disabling domain.

Table 6 shows the multivariate logistic regression model. An increase in the severity score of associated menstrual symptoms was a significant predictor of severe dysmenorrhea (OR = 1.058, 95% CI: 1.075–1.265). Age, family history of dysmenorrhea, and the MAS disabling domain were not statistically significant predictors (*P* > 0.05).

**Table 6:** Predictors of severe dysmenorrhea among the studied Egyptian disabled girls in Asyut City, Upper Egypt (Academic year 2021/2022)

Variable	Odds ratio	95% CI	*P*-value
Age in years	1.246	0.984: 1.579	0.068
Family history (No)	Reference group		
Family history (Yes)	3.279	0.971:11.076	0.056
Associated symptoms score	1.058	1.028: 1.088	0.001*
Menstruation as a disabling score	0.945	0.885: 1.010	0.094

Adjusted logistic regression of severe dysmenorrhea

*Statistically significant

## Discussion

Menstrual health is essential to the well-being and empowerment of adolescent girls. Although menstruation is a normal and healthy part of life, in some societies, the experience may be hampered by prevailing discriminatory sociocultural norms. Adolescent girls often face difficulties in managing their menstrual blood flow [27]. These challenges are more pronounced for disabled girls, who endure additional burdens of shame and stigma stemming from their gender, disability, menstruation, and the social isolation that often accompanies them [28].

Access to information and awareness about menstruation is crucial in helping disabled female adolescents manage their menstruation independently and with dignity. This, in turn, promotes their self-confidence, bodily autonomy, and realization of reproductive health and rights [8]. Mothers are commonly reported as the primary source of menstrual hygiene and dysmenorrhea information for adolescent girls, whether they are disabled or not [29–32]. Consistent with this, the current study found that mothers and friends were the most frequently reported sources of information on dysmenorrhea among the studied disabled girls.

In the present study, most disabled girls (87.1%) had poor knowledge of dysmenorrhea. By contrast, non-disabled girls generally demonstrate higher levels of knowledge about menstruation and menstrual hygiene [31, 33]. This highlights the vulnerability of disabled adolescents, who may have limited access to accurate information and often rely on nearly uneducated mothers. This underscores the need to educate and support mothers, equipping them with accurate information to pass on to their daughters—particularly those with disabilities.

According to the WaLIDD scale, all disabled girls in the present study suffered from some degree of dysmenorrhea. By comparison, only 47.6% of visually impaired women in Turkey reported menstrual pain [29]. However, comparisons are limited due to a lack of quantitative studies on dysmenorrhea in disabled populations; most prior research in this area is qualitative [14, 34].

Dysmenorrhea is also a significant issue among non-disabled girls in Egypt and worldwide. In Upper Egypt, 92% of rural adolescent girls reported dysmenorrhea [30]. Other studies report primary dysmenorrhea prevalence rates of 86.5% in Kuwaiti high school students [20], and 83% in Singaporean adolescents [35]. Similarly, university students in Turkey (83%), Saudi Arabia (87.7%), and Palestine (85.1%) reported high rates [15, 16, 36].

Approximately 70% of the studied disabled girls reported using strategies to reduce menstrual pain, with herbal drinks and analgesics being the most common methods. Nearly one-quarter missed school days due to menstrual pain. Similarly, dysmenorrhea and its related symptoms negatively affect the quality of life of non-disabled young females, primarily by contributing to school or work absenteeism. Many rely on herbal drinks and analgesics to relieve symptoms, allowing them to continue daily activities and improve quality of life [16, 20, 30, 36, 37].

Despite the high prevalence and impact of dysmenorrhea, many adolescents do not seek medical care. In this study, only 11.8% of girls visited a clinic to manage their menstrual pain, similar to findings in other contexts [32].

Primary dysmenorrhea tends to decrease with age [30, 32, 37]. However, in the current study, age was not significantly associated with dysmenorrhea severity. This may be due to the narrow age range of the study participants.

Family history of dysmenorrhea is a known predictor of its occurrence [21, 32, 38]. In this study, while a positive family history was associated with severe dysmenorrhea, it did not reach statistical significance in the adjusted model (AOR = 3.279, *P* = 0.056). This may be because all participants in the study had dysmenorrhea, and the regression model examined severity rather than presence of the condition.

Menstrual-associated symptoms such as general ache, dizziness, fatigue, suffocation, backache, and headache were commonly reported by the studied girls, similar to findings among non-disabled adolescents [21, 29, 30, 36]. A higher score for these symptoms was significantly associated with severe dysmenorrhea (OR = 1.058, 95% CI: 1.075–1.265), which may reflect higher levels of prostaglandin F2α secretion [12].

While poor menstrual knowledge is associated with poor hygiene practices [31, 38], its relationship with pain perception is unclear. In this study, knowledge about dysmenorrhea was not significantly related to pain severity. This finding aligns with studies among Taiwanese nurses [39], though it differs from findings in Malaysia, where poor knowledge was linked to increased reporting of pain [38].

Attitudes toward menstruation also influence psychosocial well-being [40]. In this study, attitude did not significantly impact the perception of severe dysmenorrhea. However, other studies have found that negative attitudes—viewing menstruation as debilitating, bothersome, or predictable—are more common among those with dysmenorrhea [39, 40]. The difference in findings may be due to the focus on severity in this study and the unique challenges faced by adolescents with disabilities.

### Limitation of the study

This study was limited to disabled girls enrolled in governmental schools. There is a need to explore the reproductive health needs of girls who face the double burden of disability and lack of educational access. The findings are also limited to blind and deaf girls in Upper Egypt. A more comprehensive evaluation with a representative sample from different governorates is needed to identify service gaps and guide future reproductive health interventions.

Due to the cross-sectional study design, comparisons between disabled and non-disabled girls could not be standardized. Moreover, causal relationships could not be established.

## Conclusions and recommendations

All studied disabled girls experienced varying degrees of dysmenorrhea. Increased severity of menstrual-associated symptoms was significantly associated with perception of severe dysmenorrhea. Disabled girls remain a vulnerable and underserved population. Efforts must be made to improve their knowledge of menstrual care and empower them with tools for proper pain management.

Educational health programs tailored to the needs of blind and deaf girls should be developed. These programs must use appropriate communication methods, such as tactile materials and sign language. Mothers—identified as key sources of information—should also be included in reproductive health education efforts.

## Data Availability

The dataset of the present study is available and could be requested from the corresponding author on reasonable reasons.
